# Effect of Ferric Carboxymaltose Supplementation in Patients with Heart Failure with Preserved Ejection Fraction: Role of Attenuated Oxidative Stress and Improved Endothelial Function

**DOI:** 10.3390/nu14235057

**Published:** 2022-11-28

**Authors:** Annachiara Mollace, Roberta Macrì, Rocco Mollace, Annamaria Tavernese, Micaela Gliozzi, Vincenzo Musolino, Cristina Carresi, Jessica Maiuolo, Martina Nicita, Rosamaria Caminiti, Sara Paone, Francesco Barillà, Maurizio Volterrani, Vincenzo Mollace

**Affiliations:** 1 Institute of Research for Food Safety & Health (IRC-FSH), Department of Health Science, University Magna Graecia, 88100 Catanzaro, Italy; 2 IRCCS San Raffaele Pisana, 88163 Rome, Italy; 3 Laboratoy of Pharmaceutical Biology, IRC-FSH Department of Health Sciences, University “Magna Græcia” of Catanzaro, Campus Universitario di Germaneto, 88100 Catanzaro, Italy; 4 Department of Systems Medicine, Tor Vergata University, 00133 Rome, Italy

**Keywords:** iron deficiency, heart failure, HF with preserved ejection fraction, ferric carboxymaltose, ferritin, transferrin saturation, 6 min walking test, EndoPAT analysis, serum malondialdehyde

## Abstract

Both clinical and experimental evidence shows that iron deficiency (ID) correlates with an increased incidence of heart failure (HF). Moreover, data on iron supplementation demonstrating a beneficial effect in subjects with HF have mostly been collected in patients undergoing HF with reduced ejection fraction (HFrEF). Relatively poor data, however, exist on the potential of iron supplementation in patients with HF with preserved ejection fraction (HFpEF). Here, we report on data emerging from a multicentric, double-blind, randomized, placebo-controlled study investigating the effect of IV supplementation with a placebo or ferric carboxymaltose (FCM) on 64 subjects with HFpEF. ID was detected by the measurement of ferritin levels. These data were correlated with cardiac performance measurements derived from a 6 min walking test (6MWT) and with echocardiographic determinations of diastolic function. Moreover, an EndoPAT analysis was performed to correlate cardiac functionality with endothelial dysfunction. Finally, the determination of serum malondialdehyde (MDA) was performed to study oxidative stress biomarkers. These measurements were carried out before and 8 weeks after starting treatment with a placebo (100 mL of saline given i.v. in 10 min; *n* = 32) or FCM at a dose of 500 mg IV infusion (*n* = 32), which was given at time 0 and repeated after 4 weeks. Our data showed that a condition of ID was more frequently associated with impaired diastolic function, worse 6MWT and endothelial dysfunction, an effect that was accompanied by elevated MDA serum levels. Treatment with FCM, compared to the placebo, improved ferritin levels being associated with an improved 6MWT, enhanced cardiac diastolic function and endothelial reactivity associated with a significant reduction in MDA levels. In conclusion, this study confirmed that ID is a frequent comorbidity in patients with HFpEF and is associated with reduced exercise capacity and oxidative stress-related endothelial dysfunction. Supplementation with FCM determines a significant improvement in diastolic function and the exercise capacity of patients with HFpEF and is associated with an enhanced endothelial function and a reduced production of oxygen radical species.

## 1. Introduction

Iron deficiency (ID) represents an emerging issue for public health and is correlated with many disease states, including myocardial dysfunction [1,2]. To date, epidemiological data have confirmed that 50% of anemia cases may be clearly correlated with ID [3,4], representing the most relevant basis for this nutritional deficiency state. 

The development of ID with or without anemia occurs as a consequence of different pathophysiological conditions [5,6], as well as malabsorption that correlates with chronic degenerative diseases [7,8]. Moreover, a condition of inflammation and oxidative stress in HF associated with inappropriate iron absorption has been found to contribute to reduced iron stores [1,2,9].

While the correlation between ID and myocardial dysfunction has clearly been shown, the potential for iron supplementation in counteracting myocardial damage and its consequences is still controversial.

Evidence exists that IV iron supplementation produces benefits in HF [10,11,12]. In particular, clear benefits of IV administration of ferric carboxymaltose (FCM) in patients suffering from HF and ID, with and without anemia, have been found with the study FAIR-HF [13]. In particular, the study showed that IV administration of a single bolus of FCM in patients with ID and HF with an ejection fraction < 40–45% produces an improvement in exercise capacity, independent of the presence of anemia [13].

These results have also been confirmed by results emerging from the CONFIRM-HF study [14], thus showing that iron supplementation improves the quality of life (QoL) and reduces the rate of hospitalization of patients with HF with reduced ejection fraction (HFrEF), with similar adverse effect and mortality compared to placebo. Limited data, however, exist on iron supplementation and HF with preserved ejection fraction (HFpEF), which represents an emerging issue in patients with metabolic disorders [15,16].

Several studies have shown that iron-deficient HFpEF patients suffered a similar impact as the health-related QoL of HFrEF patients [17,18]. Furthermore, in both HFrEF and HFpEF, concomitant ID indicates the same prognosis in terms of increased mortality from all causes, regardless of the presence or absence of anemia [19,20]. Against this epidemiological finding, there is a lack of evidence of better results after intravenous iron therapy. In a single-blind, randomized clinical trial, the anemia in HFpEF patients was treated with epoetin alfa or oral iron and was reported to have no effect on the left-diastolic ventricular volume and left ventricular mass; furthermore, no improvement in submaximal exercise capacity or QoL was observed [21]. In addition, in confirmation of these results, Kasner et al. showed no association between functional iron deficiency and exercise capacity in patients with HFpEF [22]. Finally, a condition of endothelial dysfunction seems to potentially contribute to the pathophysiological mechanisms underlying HFpEF, though its role in producing altered heart function needs to be better clarified [23,24].

In this context, this study aimed to verify the possible impact of IV administration of FCM on cardiac performance and on reliable indicators of diastolic function in patients with HFpEF. This was correlated with oxidative stress and endothelial function, which represent reliable biomarkers of vascular impairment occurring at the early stages of myocardial dysfunction in subjects in which HFpEF and ID occur simultaneously.

## 2. Materials and Methods

### 2.1. Study Design

**Figure 1:**
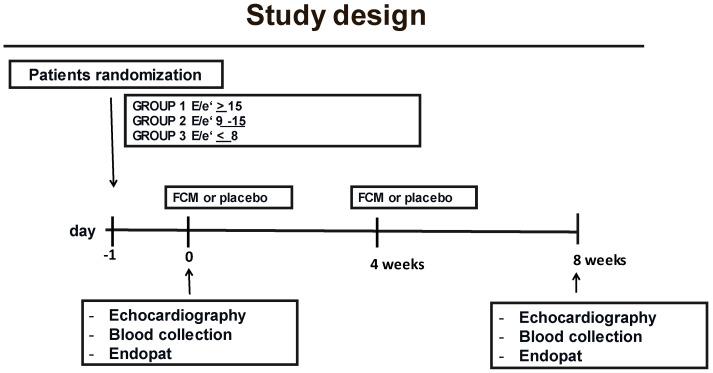
Study design. The multicentric, double-blind, randomized, placebo-controlled study was conducted on 64 subjects suffering from HFpEF classified according to the ESC Guidelines 2021.

A multicentric, double-blind, randomized, placebo-controlled study was conducted on a population of 64 subjects suffering from HFpEF classified according to the ESC Guidelines 2021 [25] and, in particular, echocardiographic parameters associated with normal ejection fraction, including left atrium (LA) size (as expressed by LA volume index, LAVI, >32 mL/m^2^), mitral E velocity > 90 cm/s, septal velocity < 9 cm/s, E/e’ ratio > 9, which have been shown to better correlate with an increased mortality rate (Figure 1).

In this cohort of patients, the prevalence, incidence, and impact of ID were compared with the degree of diastolic function impairment, reactive vasodilatation (as detected via EndoPAT measurements), and oxidative stress serum biomarkers, respectively. In particular, patients were divided into three groups according to their diastolic function as assessed by the evaluation of the E/e’ ratio (by means of echocardiography at time 0; see Section 2.2 for the methodology): Group 1: E/e’ ratio ≥ 15 [*n* = 24]; Group 2: E/e′ ratio between 9 and 14 [*n* = 24]; Group 3: E/e’ ratio ≤ 8 [*n* = 16].

The groups of patients studied were fairly homogeneous in age (69.7 ± 9.4 years) and sex (m/f ratio in the 3 groups 34/30), with the presence of metabolic syndrome in 70% of the subjects enrolled and moderately overweight. A proportion of 44% had moderate arterial hypertension on treatment with ACE-i/ARB and/or beta-blockers. Patients with valvular pathology or atrial fibrillation were excluded from the study. None of the patients were smokers at the time of the study (Table 1).

In addition, the effect of IV administration of the placebo (100 mL of saline given i.v. in 10 min) or ferric carboxymaltose (10 mL of Ferinject corresponding to 500 mg iron diluted into 100 mL of saline given i.v. in 10 min) infused at time 0 and after four weeks was evaluated. The outcome of the treatment was verified 8 weeks after starting the administration of FCM. All the patients completed the study. Moreover, the use of an iron-containing supplement was not allowed either before or during the study.

The patients were enrolled at the Metabolic Diseases Outpatient Clinic of the IRC-FSH Center of the “Magna Graecia” University of Catanzaro, in collaboration with the UTIC B of the Umberto I Polyclinic in Rome and the Unit of Cardiology at IRCCS San Raffaele, Rome. All subjects provided written informed consent at the time of enrollment, and the protocol was approved by the local ethics committee and met all the principles of the Declaration of Helsinki. The protocol is registered on the ISRCTN registry (study ID. ISRCTN12833814). 

A condition of ID was defined on the basis of serum ferritin levels < 100 nanograms/mL or with levels between 100 and 299 µg/L in the presence of transferrin saturation (TSAT) < 20% [26]. The degree of anemia was defined in the presence of Hb levels < 13 g/dL in men and <12 g/dL in women. In addition, a 6 min walking test (6MWT) was performed as described by Guyatt et al. [26]. In particular, in a long unobstructed corridor, using standardized patient instructions, patients covered as much distance as possible during the allotted 6 min. The total distance covered was measured and recorded. Staff overseeing the 6MWT were unaware of the hemodynamic results.

These determinations were performed at time 0 and at 8 weeks after starting the study. Care was taken that patients maintained the same dietary regimen during the observation period. In analogy with the sample, on the day of the visit at time 0 and after 8 weeks, for the definition of the degree of diastolic function, the patients had an echocardiographic examination, took a vascular reactivity test using the EndoPAT method, and had an additional sample taken for the determination of the levels of serum malonyldialdehyde (MDA).

### 2.2. Echocardiographic Evaluation of Diastolic Function

The diastolic function parameters used for detecting HFpEF were represented by the mitral E velocity, E/e’ ratio, and left atrium volume. In particular, E velocity is detected transmitrally. The transmitral PW spectrum includes two velocity peaks generated by differences in the atrioventricular pressure during the diastolic phase of the cardiac cycle. The first peak (wave E) corresponds to rapid ventricular filling, the one observed when, following isovolumetric relaxation, the atrial pressure far exceeds the ventricular pressure, generating an acceleration in the flow in this sense. The extent of the E peak depends on ventricular relaxation, left atrial pressure, and ventricular protodiastolic elastic return; these parameters determine the faster or slower speed of rapid filling.

The speed with which the atrio-ventricular pressure gradient tends to cancel itself following the first phase of diastole influences the speed and deceleration time (DT) of the E wave. The faster the ventricular pressure increase in protostole, the slower the deceleration time of the E wave.

Tissue Doppler (TD) is a method that has been used to assess the speed of movement of the myocardial wall. The study of diastolic function using this technique is based on the assessment of the displacement speed of the mitral annulus, with image acquisitions through the apical projection in four chambers.

The standard pattern of pulsed TD at the level of the mitral annulus includes three components: a positive (S) systolic wave, that is, directed toward the apex, and two diastolic waves, one early protodiastolic (e’) and the other late (a’) during atrial systole. Taken together, these rates and time intervals provide quantitative information on systolic longitudinal ventricular function and diastolic function. 

Compared to the transmitral flow Doppler, the pulsed annular TD is less dependent on variations in ventricular preload. For this reason, the calculation of the E/e’ ratio is a reliable parameter for the non-invasive estimation of filling pressures.

An E/e’ ratio of less than 8 is often associated with a normal filling pressure, while a ratio above 15 is observed in the case of increased filling pressures. Values between 8 and 15 need further investigation to be interpreted correctly. 

Left Atrial (LA) volume was measured from standard apical 2- and 4-chamber views at end-systole. LA borders were traced using planimetry. The borders consisted of the walls of the left atrium and a line drawn across the mitral annulus. Attention was given to bridge the ostia of pulmonary veins (when visualized) to not include the veins in the measurement. If seen, the LA appendage was excluded from measurement. The biplane method of disks was used to calculate LA volume, and measurements were rounded to the nearest integer. Left Atrial Volume Index (LAVI) was calculated by dividing LA volume by body surface area and expressed as ml/m^2^.

### 2.3. Determinations of Endothelial Function

Endothelial function was assessed using the EndoPAT 2000 technique, which measures PAT using the reactive hyperemia index (RHI, arbitrary units). Briefly, the examination was performed after 20 min of rest in a chair tilted at an angle of approximately 45° at room temperature, with a blood pressure cuff placed on the non-dominant upper arm (study arm), while the other arm served as a control. The hands were placed on the chair stands with the palms facing down so that the fingers hung freely.

The EndoPAT probes were then placed on the tip of each index finger of both hands. The probes were prevented from touching any other fingers or objects and were then electronically inflated. The PAT signal was continuously recorded on a personal computer during the test. The baseline pulse width was measured from each fingertip for five minutes. After a five-minute baseline recording on each arm, the arterial flow was then stopped in the experimental arm by rapidly inflating the cuff to the occlusion pressure of 200 or 60 mmHg plus systolic blood pressure (whichever was greater). After five minutes of occlusion, the cuff pressure was rapidly reduced, and post-occlusion recording continued for an additional five minutes in the experimental and control arms. The pulse width response to hyperemia was automatically calculated from hyperemia in the finger of the experimental arm as a ratio of post-deflation mean pulse ingestion to obtain the RH–PAT ratio or PAT ratio. EndoPAT 2000 measured not only endothelial function with the RHI but also assessed arterial stiffness by measuring the peripheral augmentation index (PAIx) from the radial pulse wave analysis. PAIx is automatically calculated as the ratio of the difference between the early and late systolic peaks of the waveform to the early and late systolic peak of the waveform related to the systolic peak, expressed as a percentage.

### 2.4. Measurements of MDA in Patients with HFpEF

Oxidative stress was assessed by quantifying the reactivity of thiobarbituric acid (TBA) as MDA in a spectrophotometer. To 0.5 mL of serum, 0.5 mL of 30% trichloroacetic acid (TCA) (Merck, Rahway, NJ, USA) was added and centrifuged at 3000 rpm for 5 min, and the supernatant was collected. Subsequently, 0.5 mL of supernatant was added to 0.5 mL of 1% TBA (Merck) in a boiling water bath for 30 min, after which the tubes were kept in an ice water bath for 10 min. The resulting absorbance of chromogen was determined at a wavelength of 532 nm at room temperature with respect to the blank reference. The concentration of MDA was read from the standard calibration curve plotted using 1, 1, 3, 3 “tetra-ethoxy propane (TEP). The amount of lipid peroxidation was expressed as MDA (µg/dL)”, using a molar extinction coefficient for MDA of 1.56 × 105 M^−1^ cm^−1^ [27].

### 2.5. Statistics

Data are presented as mean ± standard deviation (SD). StatView 5.0 (SAS Institute, inc., Cary, NC, USA) and the Statistical Package for the Social Sciences (SPSS version 21) were used for the statistical analyses. Analysis of variance (ANOVA), followed by post hoc comparisons, Student’s unpaired *t*-test, Fisher’s exact test, Pearson’s simple regression and logistic regression were used as appropriate. A two-tailed *p*-value < 0.05 indicated statistical significance.

## 3. Results

The study, conducted on 64 patients with HfpEF, documented, at baseline, a condition of ID found in 38/64 patients corresponding to 53.1% of the total subjects entering the study (Table 2). The population of patients with ID showed, under basal conditions, a moderate prevalence of male subjects (21 M vs. 17 W). On the other hand, no significant differences in BMI, Fasting Serum Glucose, serum triglycerides, LDL cholesterol, HDL cholesterol, creatinine, and Left Ventricle Ejection Fraction were found among patients with and without ID (Table 2), and treatment with Placebo or FCM did not produce any change in these parameters. However, treatment with FCM, compared to placebo, improved diastolic function indexes in patients with ID. In fact, the mean values of E/e’ ratio and LAVI were improved (from 14.1 ± 2.1 to 10.2 ± 2.5 and from 38.1 ± 12.8 to 31.2 ± 10.7, respectively; Table 2). 

Moreover, the distribution of patients into three groups made according to the degree of diastolic dysfunction (as detected on the basis of E/e’ ratio) was, overall, consistent with the data of the most recent literature on this area of interest [28,29]. In fact, the patients with severe diastolic dysfunction (Group 1; E/e’ < 8) were 24/64, equal to 31%, while the subjects enrolled in Groups 2 and 3, with a medium or low degree of dysfunction, were 24/64 and 16/64, equal to 31 and 25%, respectively (Table 1 and Table 3). On the other hand, patients in Group 1 showed, at baseline, lower ferritin levels as compared with Groups 2 and 3, thereby confirming that a condition of ID is associated, in patients with HFpEF, with an impairment in cardiac diastolic functionality (Table 3). This effect was associated with reduced exercise capacity as expressed by reduced distance covered during 6MWT, an effect accompanied by the highest serum concentration of MDA, and by altered parameters of endothelial functionality obtained via EndoPAT analysis (Table 3 and Table 4). Thus, patients with HFpEF and marked diastolic impairment showed elevated ID-associated oxidative stress and endothelial dysfunction. 

According to the degree of diastolic impairment, administration of FCB i.v. at time 0 and after 4 weeks from entering the study was able to improve ID levels, oxidative stress, and impaired endothelial function, compared to the placebo. Indeed, FCM restored the ferritin levels in patients with HFpEF associated with high degree diastolic dysfunction, an effect associated with improved cardiac performance as showed by the better response at the 6MWT (Table 3). In addition, this effect was associated with a reduction in MDA levels (Table 3) and with an enhanced endothelial-dependent response, as studied by means of the EndoPAT procedure (Table 4). 

## 4. Discussion

Our data show, for the first time, that supplementation with FCM administered via i.v. infusion in such a way as to bring ferritin within physiological limits in a cohort of patients affected by HFpEF leads to an improvement in cardiac performance evaluated by means of a 6MWT. This improvement was mainly found to occur in patients in which diastolic function was particularly compromised (Group 1 of patients enrolled in the study), as assessed by the Echo cardio color Doppler study and in particular through reliable indicators such as the E/e’ ratio and LAVI.

Moreover, the study showed that the improvement in cardiac performance, seen after supplementation with IV FCM, was associated with a marked improvement in endothelial function and in the degree of oxidative stress that characterized the basal conditions in patients at the time of their enrollment in the study.

In particular, at 8 weeks after the administration of FCM (500 mg/i.v. as a single bolus at time 0, repeated after 4 weeks), there was a marked improvement in vascular reactivity through the EndoPAT study, an effect accompanied by a significant reduction in MDA levels, a reliable index of the level of lipid peroxidation and, finally, of oxidative stress.

Diastolic dysfunction is known to represent the pathophysiological mechanism characterizing the more severe forms of HFpEF [27,30]. In particular, the reduced ability to release myocardial fibers in diastole, even in the presence of a preserved contractile capacity, determines a reduction in cardiac performance as a whole, which is associated with the basal symptoms of HF (moderate exertional dyspnea, signs of pulmonary congestion, perimalleolar edema, etc.), clearly evident at the time of the execution of the 6MWT. This condition has pathophysiological bases very different from the contractile compromise that is found in conditions of HF with low or moderately reduced EF. In fact, the condition of HF consequent to ischemic or toxic events that reduce the mass of contractile myocardium, useful for maintaining a good hemodynamic balance, is to be ascribed to a loss (often due to apoptosis) of the mass of efficient contractile myocardium or to its dysfunction, due to an impairment in the functionality of myofibrils (primary or secondary).

Conversely, diastolic dysfunction seems to have its pathophysiological origin in the progressive loss of the “lusitropic” properties of the myocardium, associated with progressive fibrosis rather than with the absolute or relative loss of the contractile myocardium [30].

The pathophysiological bases of this condition are, to date, not fully clarified. In fact, the “phenotype” of the early stages of the pathology associated with HFpEF still remains to be defined, during which the development of myocardial compromise is resolved toward an impairment of the lusitropic properties of the myocardium rather than toward the cellular apoptosis of cardiomyocytes.

However, recent studies make it possible to identify the “*primum movens*” of this pathophysiological evolution in mitochondrial oxidative stress associated with impaired iron metabolism and endothelial dysfunction, leading, in the late stages, to myocardial impairment. In particular, Zhang et al. showed that in the heart tissue of patients with HF, myocardial iron concentration was lower compared to that detected in non-failing hearts [28]. This effect was associated with the suppression of the respiratory chain and Krebs cycle enzymatic activities, which had a significant correlation with depleted iron stores, an effect that was accompanied by an increased concentration of MDA, thus showing that a condition of oxidative stress is associated with ID. This was also combined with decreased antioxidant enzymes in the myocardial tissue of ID hearts, as shown by reduced superoxide dismutase and glutathione peroxidase. Finally, they also found that iron uptake is impaired in cardiomyocytes, as expressed by decreased translocation to the sarcolemma, while the transmembrane fraction of ferroportin positively correlated with cardiac impairment. Thus, ID associated with HF seems to correlate with mitochondrial dysfunction and oxidative damage [29,31]. This fits very well with our data. In particular, the consistent reduction in ferritin levels. The reduction found in patients with a consistent reduction in diastolic function and reduced exercise capacity is associated with elevated levels of MDA, thus confirming that oxidative stress is associated with HFpEF. This effect was counteracted by restoring ferritin levels via FCM administration, thereby leading to improved exercise capacity, as found with improved performance by means of the 6mWT found after FCM treatment.

Our data also show that endothelial functionality contributes to the pathophysiology of ID-associated disorders and HFpEF. Indeed, FCM administration was able to restore altered ferritin levels and exercise capacity in patients with HFpEF and produced an improvement in endothelial-dependent vasodilatation as investigated by EndoPAT analysis in our patients at time 0 and after FCM treatment. In fact, a lower capacity to respond to flow restriction with a vasodilatory response was found in Group 1, in which a significant correlation between ID and diastolic dysfunction was found. In this group of patients, restoring ferritin levels via FCM was accompanied by improvement in all the EndoPAT parameters of endothelial-mediated vasodilatation, showing that endothelial dysfunction has a crucial role in ID-related HFpEF. This correlates with evidence suggesting that endothelial impairment accompanies HFpEF [29,31,32,33]. In particular, evidence was provided that a reduction in the number of endothelial progenitor cells (EPCs) may be found in patients with HFpEF, thereby showing that the repair of endothelial functionality may represent a potential benefit in such a class of patients. On the other hand, recent evidence in women with severe ID status suggests that there is a possible impairment in endothelial function and oxidative stress mediated by altered HDL lipoproteins, which is counteracted by i.v. iron administration; however, the mechanism of action needs to be better assessed.

In conclusion, this study confirmed that ID is a frequent comorbidity in patients with HFpEF and is associated with reduced exercise capacity and oxidative stress-related endothelial dysfunction. Supplementation with FCM determines a significant improvement in the cardiac performance of patients with HFpEF enrolled in this study, an effect that was associated with enhanced endothelial function and reduced production of oxygen radical species. Further studies, however, based on a larger number of patients, are required in order to better clarify the potential for iron supplementation in patients with HFpEF.

## Figures and Tables

**Table 1:** Distribution of 64 patients entering the study among the different study groups.

Study Groups	Without ID *n* = 26		With ID *n* = 38	
	Placebo	FCM	Placebo	FCM
Group 1 E/e’ ≥ 15	3	3	9	9
Group 2 E/e’ 9–16	5	5	7	7
Group 3 E/e’ ≤ 8	5	5	3	3

**Table 2:** Basal levels of principal biomarkers of metabolic balance and the demographics of patients with HFpEF (either with or without ID) enrolled in the study. Table also shows data on the effect of treatment with placebo and FCM.

Parameter	Without ID *n* = 26	With ID *n* = 38
**Sex (M/W)**	13/13	21/17
**Age (years)**	61.9 ± 8.9	63.2 ± 9.6
**BMI (Kg/m^2^)** Basal Placebo FCM	27.5 ± 5.6 27.2 + 4.6 26.8 + 5.1	26.5 ± 5.2 26.8 + 5.0 26.6 + 4.5
**FSG (mg/dL)** Basal Placebo FCM	114 ± 11.8 110 ± 8.1 110 ± 8.9	115.8 ± 11.2 112 ± 9.4 112 ± 10.1
**Triglycerides (mg/dL)** Basal Placebo FCM	195 ± 21.5 190 ± 18.4 191 ± 16.9	196 ± 21.8 192 ± 15.6 190 ± 17.8
**Creatinine (mg/dL)** Basal Placebo FCM	1.0 ± 0.9 1.1 ± 0.7 1.0 ± 0.8	1.1 ± 0.8 1.0 ± 0.7 1.0 ± 0.9
**LDL-Cholesterol (mg/dL)** Basal Placebo FCM	94.2 ± 23.8 95.1 ± 21.2 94.2 ± 20.6	96.1 ± 24.3 95.4 ± 24.2 95.2 ± 21.9
**HDL-Cholesterol (mg/dL)** Basal Placebo FCM	38.0 ± 6.8 40.2 ± 6.5 39.7 ± 7.1	39.1 ± 6.1 41.5 ± 6.4 42.4 ± 6.3
**LVEF (%)** Basal Placebo FCM	54.6 ± 6.0 54.9 ± 5.4 55.6 ± 5.2	53.5 ± 5.8 54.3 ± 5.5 56.4 ± 5.7
**E/e’ ratio** Basal Placebo FCM	9.2 ± 2.4 9.1 ± 2.6 8.8 ± 2.2	14.4 ± 2.7 14.1 ± 2.1 11.8 ± 2.5 *
**LAVI (mL/m^2^)** Basal Placebo FCM	37.8 ± 12.7 36.6 ± 10.3 37.4 ± 11.7	39.2 ± 14.1 38.1 ± 12.8 34.4 ± 13.7

BMI: body mass index; FSG: fasting serum glucose; LVEF: left ventricular ejection fraction; LAVI: Left Atrial Volume Index. * *p* < 0.05 Placebo vs. FCM.

**Table 3:** The effect of placebo or FCM on ferritin, TSAT, 6MWT, and MDA levels in patients with HFpEF according to their diastolic function as expressed by E/e’ ratio. Data are expressed as mean ± S.E.M.

Parameter	Group 1 E/e’ ≥ 15 *n* = 24	Group 2 E/e’ 9–14 *n* = 24	Group 3 E/e’ ≤ 8 *n* = 16
**Ferritin (ng/mL)** Basal Placebo FCM	60.3 ± 13.3 62.3 ± 12.4 129.1 ± 13.9 *	110.2 ± 14.6 112.2 ± 13.6 132.2 ± 14.3	98.2 ± 14.1 120.4 ± 14.6 145.1 ± 15.3
**6 mWT (mt/6 min)** Basal Placebo FCM	291.1 ± 22.6 294.7 + 20.7 354.4 + 23.7 *	335.4 ± 21.3 340.8 + 20.5 365.9 + 21.1	395.4 ± 28.6 401.5 + 24.4 421.6 + 24.3
**MDA (μg/dL)** Basal Placebo FCM	1.1 ± 0.1 1.0 ± 0.2 0.7 ± 0.1 *	1.3 ± 0.2 1.4 ± 0.2 0.8 ± 0.1 *	1.6 ± 0.1 1.5 ± 0.2 0.9 ± 0.2 *

6 mWT—6 min walking test; MDA–malondialdehyde; * *p* < 0.05 Placebo vs. FCM.

**Table 4:** Parameters of endothelial functionality measured via EndoPAT methodology (see methods) at time 0 (basal) and after 8 weeks from starting the treatment with FCM or the placebo in patients with HFpEF classified according to the E/e’ ratio (Groups 1, 2, and 3). Data are expressed as mean ± S.E.M.

EndoPAT Index	Group 1 E/e’ ≥ 15 *n* = 24	Group 2 E/e’ 9–14 *n* = 24	Group 3 E/e’ ≤ 8 *n* = 16
**RHI** Basal After Placebo After FCM	1.50 ± 0.3 1.54 ± 0.4 2.15 ± 0.11 *	1.90 ± 0.4 1.93 ± 0.3 2.12 + 0.4	1.95 ± 0.2 1.98 ± 0.3 2.21 ± 0.06
**fRHI** Basal After Placebo After FCM	0.20 ± 0.05 0.21 ± 0.03 0.28 + 0.04 *	0.24 ± 0.03 0.23 ± 0.02 0.24 ± 0.04	0.28 ± 0.02 0.25 ± 0.03 0.29 ± 0.03
**AI** Basal After Placebo After FCM	7.6 ± 3.2 8.1 ± 2.6 9.1 ± 4.2	8.3 ± 2.6 8.4 ± 2.3 8.4 ± 3.8	9.6 ± 2.5 9.5 ± 2.2 9.6 ± 3.5

RHI—reactive hyperemia index; fRHI—Framingham reactive hyperemia index; AI—augmentation index; * *p* < 0.05 FCM vs. placebo.

## Data Availability

The data presented in this study are available upon request from the corresponding author.
